# A Rare Coronary Artery Anomaly: Single Coronary Artery Originate From Right Sinus Valsalva R-IIP Sub-Group Type

**DOI:** 10.4021/cr185w

**Published:** 2012-05-20

**Authors:** Ferhat Ozyurtlu, Halit Acet, Mehmet Zihni Bilik, Abdurrahman Tasal

**Affiliations:** a Special Sada Hospital, Clinic of Cardiology Izmir, Turkey; b Diyarbaklr Training and Research Hospital, Department of Cardiology, Diyarbakır, Turkey; c Bezmialem Universty, Department of Cardiology, Istanbul, Turkey

**Keywords:** Coronary angiography, Coronary vessel anomalies, Sinus of Valsalva/abnormalities

## Abstract

We reported a single coronary artery case that in the R-IIP sub-group which is a rare sub-group comparing with other sub-groups of single coronary artery originating from right sinus valsalva.

## Introduction

Single coronary artery (SCA) is a rare congenital anomaly in which the entire coronary system arises from a solitary ostium. As an isolated finding, its incidence is 0.024% to 0.066% in the general population undergoing coronary angiography [1-3]. However, it is encountered more frequently with other congenital cardiac malformations such as persistent truncus arteriosus,tetralogy of Fallot, transposition of the great arteries, or pulmonary atresia. We reported a single coronary artery case that in the R-IIP sub-group which is a rare sub-group comparing with other sub-groups of single coronary artery originating from right sinus valsalva.

## Case Report

**Figure 1:**
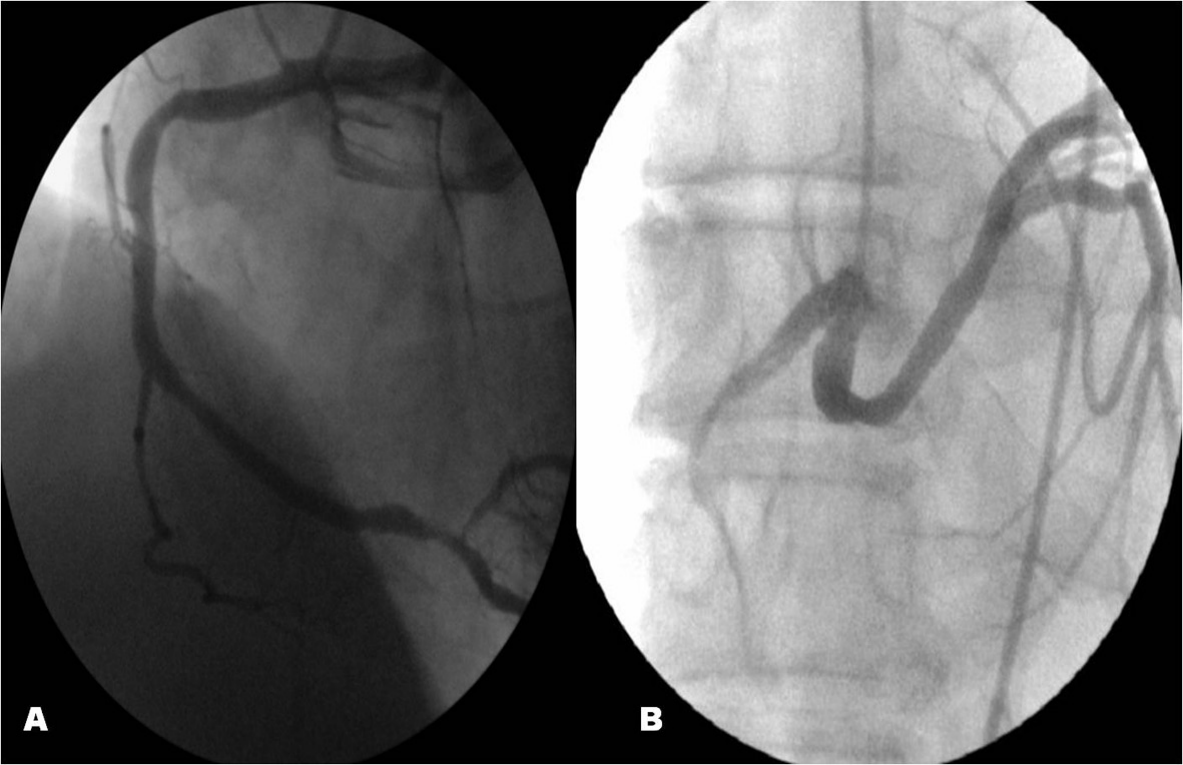
With nonobstructive stenosis in the mid portion of RCA, there were long critical stenosis that starting from osteal part of posterolateral artery.

**Figure 2:**
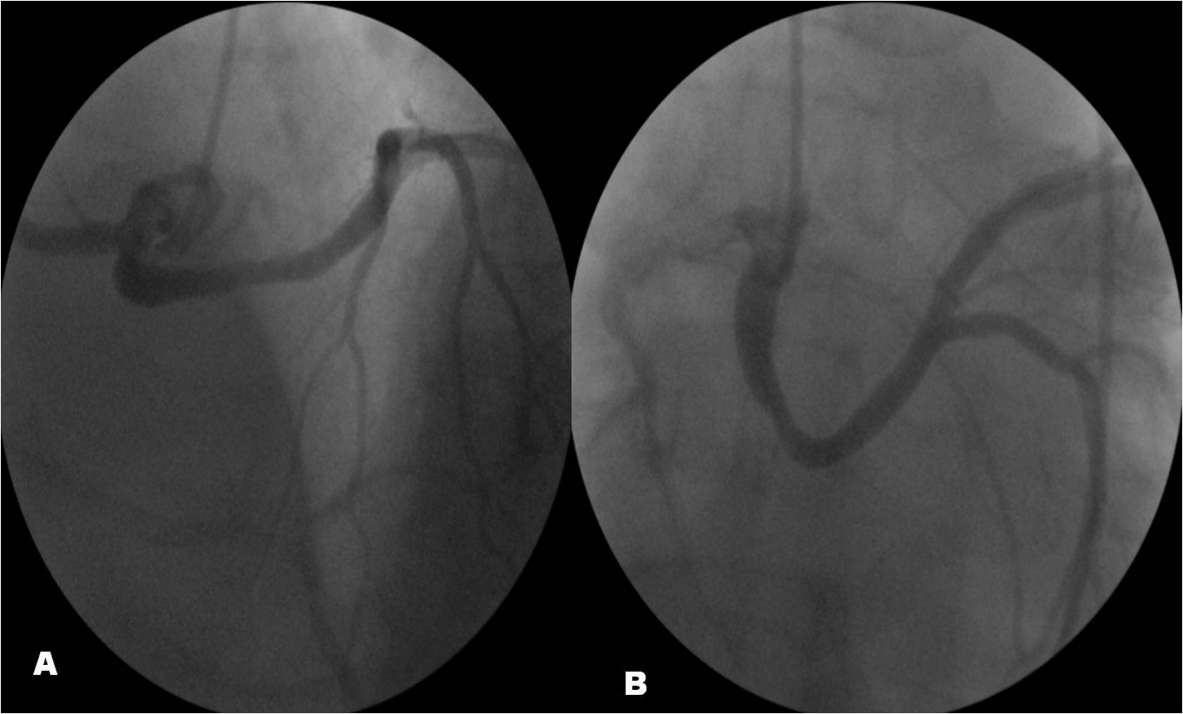
The left main coronary artery (LMCA) was leaving two branches as left anterior descending artery (LAD) and left circumflex (LCx) after traversing the base of heart. The stenosis of post-ectatic segment in the proximal LMCA was non-critical.

A 65-year-old male patient with typical chest pain was admitted to an outside medical center. After initial evaluation and treatment he was referred to our hospital for coronary angiography. Hypertension and smoking were risk factors for coronary artery disease. Physical examination was normal. There was ST depression in the inferior leads in electrocardiogram. Transthoracic echocardiography revealed only degenerative aortic valve and mild aortic insufficiency. Coronary angiography, ventriculography and aortography was performed. Left coronary system could not be imaged with left Judkins chateter. Single coronary artery originated from right sinus valsalva was shown by right Judkins chateter. Right coronary artery (RCA), was leaving after a short main coronary artery and following the normal course. With nonobstructive stenosis in the mid portion of RCA, there were long critical stenosis that starting from osteal part of posterolateral artery (Fig. 1). The left main coronary artery (LMCA) was leaving two branches as left anterior descending artery (LAD) and left circumflex (LCx) after traversing the base of heart. The stenosis of post-ectatic segment in the proximal LMCA was non-critical (Fig. 2). Medical treatment was decided for the lesion in posterolateral artery.

## Discussion

Single coronary artery is a coronary artery anomaly that describes the origin of both RCA and LMCA from a single aortic sinus. It is usually diagnosed incidentally during coronary artery angiograms or on postmortem evaluations. In a large series of 126,595 patients undergoing coronary angiography, a single coronary artery from the right sinus of valsalva was found in 0.019% [2], 40% of single coronary artery anomaly cases are associated with congenital heart diseases such as fallot tetralogy, transposition of great arteries, persistent truncus arteriosus and pulmonary atresia [4].

The current classification system of single coronary arteries was proposed by Lipton et al [1]. In this classification, single coronary artery: According to exit origine ‘R’ is the right sinus of valsalva and the ‘L’ left sinus of valsalva. According to the anatomical course of the artery, ‘I’ single coronary artery followed a normal course of the right or the left coronary artery. ‘II’ single coronary artery, after leaving the right or left coronary sinus to provide the contralateral coronary artery, crosses the base of the heart in a wide range of transverse body. ‘III’ single coronary artery, after leaving the right coronary sinus of valsalva LAD and LCx arise separately from proximal part of the artery. Finally, classified according to relation between anomalous coronary artery with the aorta and pulmonary artery,‘A’ the left main passes anterior to the pulmonary artery, ‘B’ the left main passes between the aorta and pulmonary artery, ‘P’ the left main passes posterior to the aorta. Yamakana et al. have added to this classification the ‘S’ septal (through the interventricular septum) and ‘C’ the combined type [1, 2]. Our case was type R-IIP. Although R-IIS sub-group is the most common type of R-II anomalies, R-IIP sub-group is very rare.

The single coronary artery anomaly is usually asymptomatic, but may present as myocardial ischemia, syncope or sudden cardiac death depending on its course and the presence and severity of atherosclerosis. Myocardial ischemia or sudden cardiac death are usually associated with its course between the aorta and main pulmonary artery [5]. Yet there is not a treatment strategy guide for single coronary artery [6]. The course of anomalous coronary artery and associated with coronary atherosclerotic disease determine treatment strategy. Due to the risk of sudden death in patients whose anomalous coronary artery courses between aorta and pulmonary artery, coronary artery bypass surgery is useful even if patients have not severe atherosclerotic coronary stenosis. Surgical strategy involves the replacement of anomalous coronary artery to coronary sinus appropriately or bypass surgery.

## References

[1] Lipton MJ, Barry WH, Obrez I, Silverman JF, Wexler L (1979). Isolated single coronary artery: diagnosis, angiographic classification, and clinical significance. Radiology.

[2] Yamanaka O, Hobbs RE (1990). Coronary artery anomalies in 126,595 patients undergoing coronary arteriography. Cathet Cardiovasc Diagn.

[3] Desmet W, Vanhaecke J, Vrolix M, Van de Werf F, Piessens J, Willems J, de Geest H (1992). Isolated single coronary artery: a review of 50,000 consecutive coronary angiographies. Eur Heart J.

[4] Antonellis J, Rabaouni A, Kostopoulos K, Margaris N, Kranidis A, Salahas A, Ifantis G (1996). Single coronary artery from the right sinus of Valsalva, associated with absence of left anterior descending and an ostium-secundum-type atrial septal defect: a rare combination. A case report. Angiology.

[5] Shirani J, Roberts WC (1993). Solitary coronary ostium in the aorta in the absence of other major congenital cardiovascular anomalies. J Am Coll Cardiol.

[6] Braun MU, Stolte D, Rauwolf T, Strasser RH (2006). Single coronary artery with anomalous origin from the right sinus Valsalva. Clin Res Cardiol.

